# Effect of Long-term Smoking on Whole-mouth Salivary Flow Rate and Oral Health

**DOI:** 10.5681/joddd.2010.028

**Published:** 2010-12-21

**Authors:** Maryam Rad, Shahla Kakoie, Fateme Niliye Brojeni, Nasim Pourdamghan

**Affiliations:** ^1^ Member of Kerman Neuroscience Research Center, Specialist of Oral Medicine, Kerman University of Medical Sciences, Kerman, Iran; ^2^ Member of Kerman Oral and Dental Diseases Research Center, and Assistant Professor, Department of Oral Medicine, Kerman University of Medical Sciences, Kerman, Iran; ^3^ Dentist, Private Practice, Kerman, Iran; ^4^ General Practitioner, Kerman University of Medical Sciences, Kerman, Iran

**Keywords:** Oral health, saliva, smoking, tobacco, xerostomia

## Abstract

**Background and aims:**

Change in the resting whole-mouth salivary flow rate (SFR) plays a significant role in patho-genesis of various oral conditions. Factors such as smoking may affect SFR as well as the oral and dental health. The primary purpose of this study was to determine the effect of smoking on SFR, and oral and dental health.

**Materials and methods:**

One-hundred smokers and 100 non-tobacco users were selected as case and control groups, respectively. A questionnaire was used to collect the demographic data and smoking habits. A previously used questionnaire about dry mouth was also employed. Then, after a careful oral examination, subjects’ whole saliva was collected in the resting condition. Data was analyzed by chi-square test using SPSS 15.

**Results:**

The mean (±SD) salivary flow rate were 0.38 (± 0.13) ml/min in smokers and 0.56 (± 0.16) ml/min in non-smokers. The difference was statistically significant (P=0.00001). Also, 39% of smokers and 12% of non-smokers reported experiencing at least one xerostomia symptom, with statistically significant difference between groups (p=0.0001). Oral lesions including cervical caries, gingivitis, tooth mobility, calculus and halitosis were significantly higher in smokers.

**Conclusion:**

Our findings indicated that long-term smoking would significantly reduce SFR and increase oral and dental disorders associated with dry mouth, especially cervical caries, gingivitis, tooth mobility, calculus, and halitosis.

## Introduction

There are clinical and epidemiological evidences regarding the adverse effects of tobacco on oral health.^1^Numerous studies have shown that tobacco use would lead to an increased incidence and severity of periodontal diseases and a higher rate of tooth loss. The adverse effects of cigarette smoking and other forms of tobacco are numerous and tobacco use has been associated with gingival, oral mucosa and dental alterations.^2^

Saliva is a complex and important body fluid which is very essential for oral health.^3^ Saliva is required for protecting the oral mucosa, teeth remineralization, digestion, taste sensation, pH balance and phonation. It includes a variety of electrolytes, peptides, glycoproteins, and lipids which have antimicrobial, antioxidant, tissue repair, and buffering properties.^4^ Therefore, altered whole-mouth salivary flow rate (SFR) has an important role in the pathogenesis of oral and dental diseases.^5^ Saliva is the first biological fluid that is exposed to cigarette smoke, which contains numerous toxic compositions responsible for structural and functional changes in saliva.^6^

There are also several studies concerning the effect of chewing tobacco and smoking on salivary secretion. While some of these studies have shown an increase in SFR especially in short term,^7-9^ no significant changes in tobacco users’ flow rate was reported as opposed to non-tobacco users.^3,5^

However, Bouquot & Schroeder ^8^have reported that although smoking causes a short-term increase in salivary secretion, the long-term effects of tobacco use are unclear. Intense smokeless tobacco use has been shown to result in degenerative changes of more than 40% of minor salivary glands located in the site of chronic tobacco placement.^8^ The aim of the present study was to analyze the long-term effects of smoking on salivary flow rate.

## Materials and Methods

The subjects of this study were selected from the patients referring to the Department of Oral Medicine at Kerman University of Medical Sciences School of Dentistry for routine dental care. The exclusion criteria included age over 50 years, alcohol consumption, a history of trauma to head and neck , wearing dentures, pregnant or postmenopausal women, a history of radiotherapy, and patients with systemic or salivary gland diseases or under any drug therapy. The subjects comprised individuals who had smoked cigarettes daily for more than 6 months as the case group, and non-tobacco users as the controls.^3^ Each group comprised of 100 apparently healthy adults that were matched respecting sex and age.

A questionnaire was used to collect demographic information, the subjects’ report of the existence of halitosis or unpleasant tastes, and the smoking habit (frequency and duration). A careful oral examination was performed for all subjects. In each case, changes in the oral mucosa (ulceration, erythema, keratosis, discoloration…), signs of periodontal diseases (gingivitis, mobility, bleeding on probing, clinical attachment loss), and the existence of dental caries (cervical, occlusal) were recorded.

The xerostomia level in this population was determined by asking the subjects specific questions about dry mouth, using the questionnaire designed by Fox et al.^10^ Based on this questionnaire, the positive answer to at least one of the three questions reveals subjects with salivary dysfunction. These three questions are:^10^

1. Do you sip liquids to aid in swallowing dry foods?
2. Does your mouth feel dry when eating a meal?
3. Do you have difficulties swallowing any foods?

Saliva samples were also collected from each subject. Saliva collection was performed between 9:00 am to 12:00 pm to avoid diurnal variation. Each subject was requested not to eat, drink or perform oral hygiene or smoke 60 minutes before and during the collection of saliva. The subjects were seated on the dental chair and asked to spit in a graduated container every 1 minute for 5 minutes.^11^

Data was analyzed using SPSS 15 computer software. Student’s t-test and Chi-square test were applied to assess between-group differences. A p-value of less than 0.05 was considered as statistically significant.

## Results

The case (mean age, 36.6±8.9 years) and the control (mean age, 34.5±7.9 years) groups each consisted of 96 males and 4 females. Subjects in the case groups smoked 14.8±8.30 cigarettes per day (minimum=2, maximum=40 cigarettes), and the mean duration of smoking was 12.15±6.84 years (minimum=2, maximum=30 years). Subjects who smoked more cigarettes per day and had the habit for a longer period, were at greater risk for symptoms of dry mouth (P= 0.005). Accordingly, answers to the three main questions revealed that 39% of smokers and 12% of non-smokers had symptoms of dry mouth (Table 1).

**Table 1:** Comparison of responses of smokers and non-smokers groups to dry mouth questionnaire

Question	Answer of smokers		Answer of Non-smokers		P value
	Yes	No	Yes	No	
Dose your mouth feel dry at night or on awakening?	38	65	8	92	0.0001
Dose your mouth feel dry at other times of the day?	38	62	15	85	0.0001
Do you keep a glass of water by your bed?	9	91	3	97	0.74
Do you sip liquids to aid in swallowing dry foods?	32	68	6	94	0.0001
Dose your mouth feel dry when eating a meal?	31	69	6	94	0.0001
Do you have difficulties swallowing any foods?	31	69	7	93	0.0001
Do you chew gum daily to relieve oral dryness?	12	88	8	92	0.34
Do you use hard candies or mints daily to relieve oral dryness?	4	96	6	96	0.51
					
	Too little	Too much, or don't notice	Too little	Too much, or don't notice	
Dose the amount of saliva in your mouth seem to be too little, too much, or you don’t notice it?	32	68	11	89	0.0001

Mean SFR was 1.93 ml/5 min ± 0.065 (0.38 ml/min ± 0.13) for smokers, and 2.78 ml/5 min ± 0.819(0.56 ml/ min ± 0.16) for non-smokers, with a significant statistical difference (p=0.0001).

Halitosis was reported in 55% of smokers, and 28% of non-smokers; this difference was significant (p=0.0001). Unpleasant taste after smoking was recorded in 37 smokers (28 smokers had bitter and 9 smokers had salty taste). In smokers, gingivitis (p=0.0001), mobility (p=0.0001), calculus (0.002), and cervical caries (p=0.0001) were significantly higher than non-smokers. Occlusal caries were lower in smokers compared to non-smokers, but the difference was not significant (p=0.707) (Table 2).

**Table 2:** Results of oral examination in smokers and non-smokers groups evaluated

Oral and dental diseases	Smokers	Non-smokers	P value
Cervical caries			0.0001
Yes	86	21	
No	14	79	
Occlusal caries			0.0707
Yes	82	84	
No	18	16	
Gingivitis			0.0001
Yes	82	47	
No	18	53	
Tooth mobility			0.0001
Yes	51	17	
No	49	83	
Calculus			0.002
Yes	88	70	
No	12	30	

The results showed that 74 cases had oral lesions, including erythema, white plaque, thrush and angular chellitis and were seen in 63 smokers, and 11 non-smokers (Table 3). In smokers the most common lesion was homogenous white plaque (Table 3). The most common site of oral lesions was buccal mucosa (Table 4). Although the prevalence of oral lesions in the smokers were more than that of non-smokers, the difference was not significant.

**Table 3:** Comparison of oral lesions smokers and non-smokers groups evaluated

Lesion	smokers	Non-smokers	Total
Homogeneous white plaque	26	2	28
Erythema	12	4	16
Median rhomboid glossitis	8	0	8
Pigmentation	5	3	8
Angular cheilitis	4	0	4
Non-homogeneous white plaque	2	2	4
Thrush	3	0	3
Median rhomboid glossitis and pigmentation	1	0	1
Erythema and pigmentation	1	1	1
Erythema and pigmentation and median rhomboid glossitis	1	1	1
Total	63	11	74

**Table 4:** Site of oral lesions in smokers and non-smokers groups evaluated

Site of lesions	Smokers	Non-smokers	Total
Buccal mucosa	22	3	25
Tongue	16	1	17
Gingiva	8	3	11
Lip	8	3	11
Palate	9	1	10
Tongue and buccal mucosa	2	0	2

## Discussion

Our results showed that the mean SFR in smokers was significantly lower than that in non-smokers (p=0.0001). Also, smoker subjects who experienced xerostomia symptoms were significantly more than non-tobacco users. It seems that these questions especially questions about feeling of dry mouth during eating and swallowing are very important, and can correctly indicate dry mouth.

In the present study, the mean (±SD) level of SFR was found to be 0.38 (±0.13) in smokers, and 0.56 (±0.16) in non-smokers. This finding is in contrast with the results of a study among tobacco chewers in India, where the differences in mean SFR between smokers (3.12±1.56) and non-smokers (3.40±1.69) as well as between tobacco chewers and tobacco non-chewers were not significant.^3^ A number of studies have shown that while cigarette smoking would typically cause a noticeable short-term increase in salivary flow rates, the long-term influence of tobacco use is still unclear.^8^ It has also been observed that some individuals develop tolerance to the salivary effects of smoking in the long-term use.^9^ However, our results are comparable to studies that have shown smoking is one of the risk factors for reducing saliva and xerostomia.^12-15^ It seems that smoking increases the activity of salivary glands in anyone who begins smoking, but in long-term use, it reduces SFR. In our study, the majority of smokers were heavy smokers (mean number of cigarettes per day, 14.8, the mean duration, 12.5 years). Thus, there is a significant difference in the secretion rate of saliva and dry mouth between smokers and non-smokers.

The results of our study also showed that halitosis and unpleasant taste were significantly different between smokers and non-smokers. Tobacco smoking is known to increase thresholds for the sensations of taste and smell, and both smoked and smokeless tobacco usage produce unpleasant breath odors or halitosis,^8,9^thereby, diminishing the ability to detect various tastes and smells.^8^ In other studies, however, no statistically significant difference was observed for either overall taste sensitivity or for the specific taste primaries between smokers and non-smokers.^9^ In relation to taste, a dose-related association is reported for bitter sensations, and to a lesser extent for salty tastes, but there is little change in the ability to detect sweet or sour substances,^8^ which is similar to our study. This problem becomes progressively worse with each additional year of tobacco use. Interestingly, individuals who quit smoking express a very strong desire to have sweet foods.^8^

Our results implied that gingivitis, mobility, and calculus were significantly higher in smokers than non-smokers (p=0.0001). This finding is similar to the results of several studies,^15-17^, which reported that probing depth, attachment loss, plaque index, bone loss, and calculus were higher in smokers compared to non-smokers. Contrary to this, in a study in Sweden, it was concluded that smoking is a significant risk indicator for tooth loss and probing attachment loss, while plaque index and oral hygiene were similar in smokers and non-smokers.^18^

In the present study, cervical caries were significantly higher in smokers than non-smokers (p=0.0001). Nineteenth century dental surgeons believed that smoking protected against caries. However, most recent studies have concluded that cigarette smoking is certainly associated with an increased caries rate but a cause-and-effect relationship is still not proven.^8,18,19^ These studies have shown that smokers have a significantly higher number of carious or repaired teeth than non-smokers, and heavy smokers are more affected than light smokers. Also, smokers have higher plaque rates, poorer oral hygiene habits and skills, fewer visits to dentists, and lesser overall health standards than non-smokers. Therefore, these factors may be the reasons for the increased caries rate in smokers. However, in the present study, the majority of subjects in the control group were heavy smokers, and the mean SFR in smokers was significantly lower than non-smokers. Hence, it seems that a decrease in saliva and poorer oral hygiene habits can lead to increased caries in smokers.

Salivary microbe counts are affected by many variables such as smoking. A number of studies showed that smoking has been strongly associated with higher presence of Candida species.^8,20,21^Overgrowth of Candida species can lead to oral candidiasis, that can manifest itself as erythema, white plaque, thrush, median rhomboid glossitis, and angular cheilitis.^8,22^ In our study, the most common lesion was white plaque in the buccal mucosa, which can be associated with candidiasis. Although many studies have shown that smoking is an important predisposing factor for oral candidiasis, how it may affect on oral candida is still controversial.^21^ It has been shown that the great majority (83%) of oral candidiasis patients are moderate to heavy cigarette smokers. The rate of oral candidal carriage can be variably affected by smoking, becoming either more or less intense in different individuals.^8^

## Conclusion

Our findings indicated that long-term smoking significantly reduces SFR and increases oral and dental disorders associated with dry mouth, especially cervical caries, gingivitis, tooth mobility, calculus and halitosis.
